# Ossification of the cervical anterior longitudinal ligament is an underdiagnosed cause of difficult airway: a case report and review of the literature

**DOI:** 10.1186/s12871-020-01077-9

**Published:** 2020-06-30

**Authors:** Min Xu, Yue Liu, Jing Yang, Hao Liu, Chen Ding

**Affiliations:** 1 Department of Anesthesiology, West China Hospital, Sichuan University, No.37 Guo Xue Ave, Chengdu, Sichuan 610041 PR China; 2 Department of Orthopedics, West China Hospital, Sichuan University, No.37 Guo Xue Ave, Chengdu, Sichuan 610041 PR China

**Keywords:** Ossification of the anterior longitudinal ligament, Difficult airway, Anesthesia

## Abstract

**Background:**

Ossification of the anterior longitudinal ligament (OALL) of the cervical spine is a common, but rarely symptomatic, condition mostly observed in the geriatric population. Although the condition usually requires no intervention, it could lead to a difficult airway and compromise the patient’s safety.

**Case presentation:**

Here, we describe the case of a 50-year-old man with cervical myelopathy and OALL that resulted in difficult endotracheal intubation after induction of anesthesia. Radiography and magnetic resonance imaging findings showed OALL, with prominent osteophytes involving four cervical vertebrae, a bulge in the posterior pharyngeal wall, and a narrow pharyngeal space. Airtraq® laryngoscope-assisted intubation was accomplished with rapid induction under sevoflurane-inhaled anesthesia.

**Conclusion:**

Anesthesiologists should understand that OALL of the cervical spine could cause a difficult airway. However, it is difficult to recognize asymptomatic OALL on the basis of routine airway evaluation guidelines. For susceptible populations, a thorough evaluation of the airway, based on imaging studies and a history of compression symptoms, should be considered whenever possible. In case of unanticipated difficult intubation, anesthesiologists should refer to guidelines for unanticipated difficult airway management and identify OALL of the cervical spine as the cause.

## Background

Diffuse idiopathic skeletal hyperplasia (DISH), also named as “Forestier’s disease,” is a rare idiopathic spinal disease characterized by a “flowing” ossification of the anterior longitudinal ligament (OALL) of the spine with an unknown etiology [1]. OALL of the cervical spine is common in patients over the age of 50 years, with a prevalence of approximately 15–20% in the elderly [2, 3]. Although usually asymptomatic, in rare cases, osteophytes caused by OALL of the cervical spine can encroach the digestive tract and airway, leading to swallowing and respiratory problems [4]. Regardless of the presence of symptoms, patients are at risk of developing a difficult airway after anesthesia induction due to cervical OALL [5, 6]. Here, we describe the case of a 50-year-old man with OALL of the cervical spine who underwent cervical surgery with difficult endotracheal intubation after anesthesia. Airtraq® laryngoscope-assisted intubation was accomplished under rapid induction. We also discuss our case in relation to a case-based literature review.

## Case presentation

**Fig. 1:**
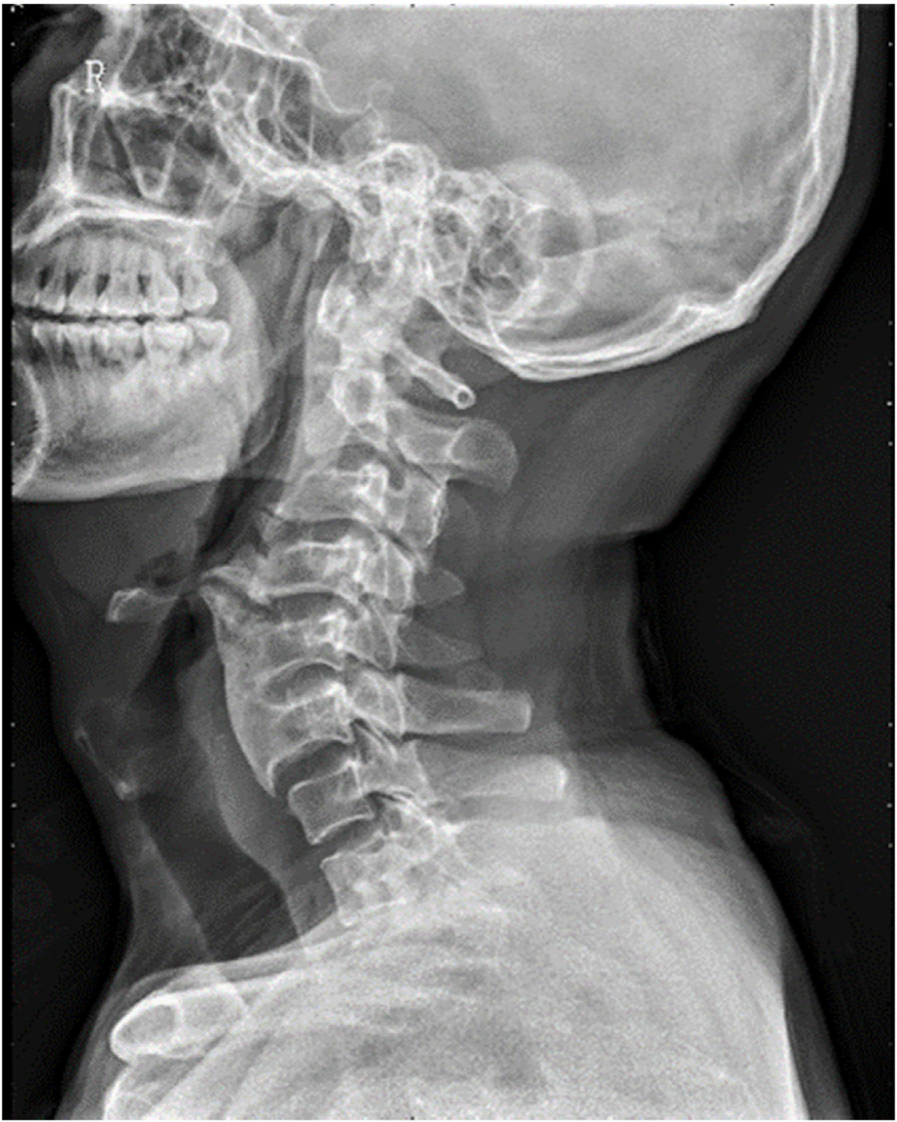
A lateral cervical spine radiograph displayed osteophyte from C3 to C6. “Beak-like” osteophyte in front of the C4 vertebrae significantly protruded forward

**Fig. 2:**
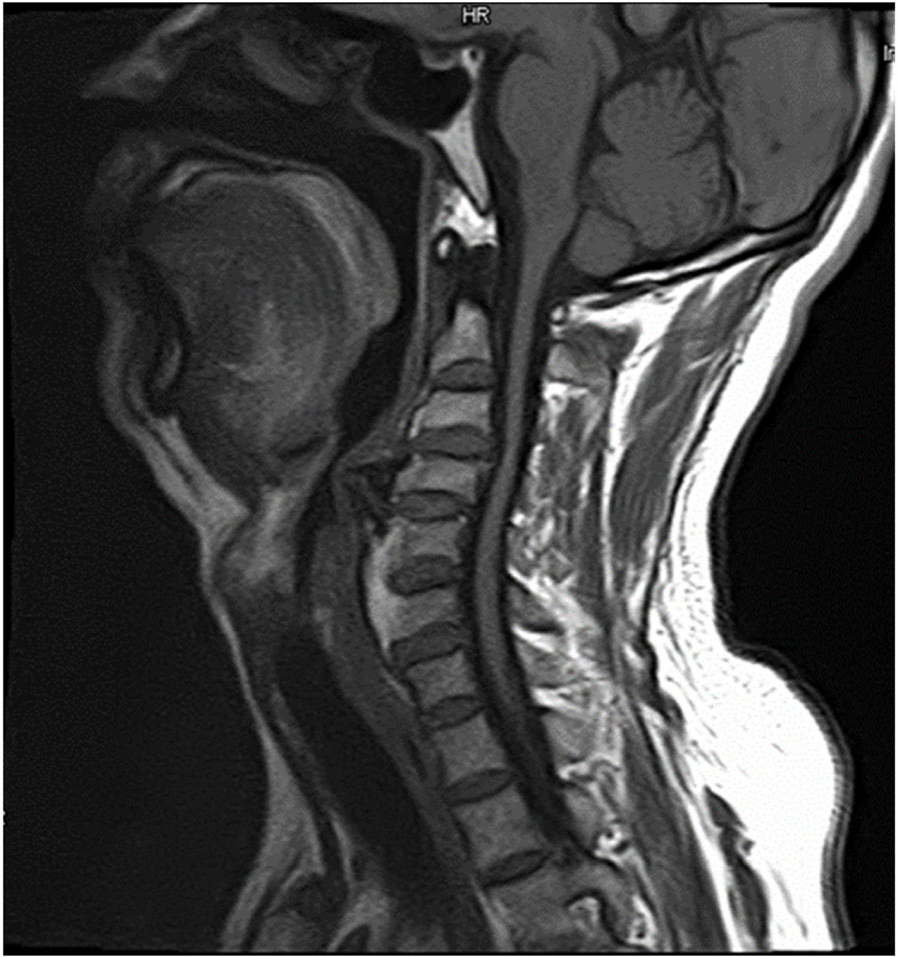
A lateral cervical spine MRI displayed osteophyte from C3 to C6. “Beak-like” osteophyte in front edges of the C4-C5 vertebrae protruded forward and compressed the esophagus and airway, and the post-protruding C3–4 disc compressed the spinal cord

A 50-year-old man (height, 165 cm; weight, 66 kg) who complained of numb hands and experienced unsteadiness while walking was diagnosed with C3–C4 intervertebral disc herniation and C3–C6 OALL. He was scheduled to undergo C3–C6 anterior cervical osteophyte resection, C3–C4 anterior discectomy, spinal canal decompression combined with interbody fusion, internal fixation, and C4–C5/C5–C6 artificial cervical disc replacement. The patient had a 30-year history of smoking and had never undergone a surgery. The preoperative evaluation showed an American Society of Anesthesiologists class II and a normal airway. The inter-incisor distance was 48 mm, which was measured using a ruler with the patient sitting in the neutral position with his mouth maximally open. The thyromental distance was 60 mm, which was measured between the prominence of the thyroid cartilage and the bony point of the chin with the head maximally extended on the neck. The patient exhibited a Mallampati Class II airway. He did not present with any limitation in neck movements (the range of neck motion included the “chin-to-chest” distance and the full extension of the head), esophageal and airway obstruction, or hoarseness. A lateral cervical spine radiograph showed a “beak-like” osteophyte in front of the C4 vertebra, which protruded forward significantly (Fig. 1). In addition, a lateral magnetic resonance image (MRI) of the cervical spine showed that the “beak-like” osteophyte compressed the esophagus and airway, while the protruding C3–C4 disc compressed the spinal cord (Fig. 2). Although there was no clinical evidences nor signs, the patient’s image finding revealed that he would have a difficult airway (at the time of ventilation and/or intubation). The patient refused our suggested awake intubation, and, therefore, we chose succinylcholine for rapid induction to prevent intubation failure and wake the patient up in time. Moreover, we prepared a fiber-bronchoscope, video laryngoscope, and small-sized endotracheal tube. The patient provided written consent for publication of this report.

After entering the operating room, the patient was carefully placed in the sniffing position and the cervical hyperextension position without any discomfort. His vital signs were normal. After pre-oxygenation and gradual induction of anesthesia through inhalation of sevoflurane, the patient was deeply sedated with spontaneous breathing. No airway obstruction (airway obstruction score [AOS], 1) was observed, and mask ventilation was easy (Han’s Mask ventilation score, 2). After spraying the throat with 2% lidocaine and administering succinylcholine and propofol, we performed direct laryngoscopy using a Macintosh blade (“adult large” size, 150 mm), which facilitated a Cormack-Lehane grade IV view. Vision was obscured by a mass approximately 1 cm in diameter in the posterior pharyngeal wall with a smooth mucosal surface. The Airtraq® video laryngoscope (Prodol Meditec, Bizkaia, Spain) was subsequently used and provided a Cormack-Lehane grade II view. Finally, successful intubation was achieved, although only the posterior margin of the glottic structure was visualized.

**Fig. 3:**
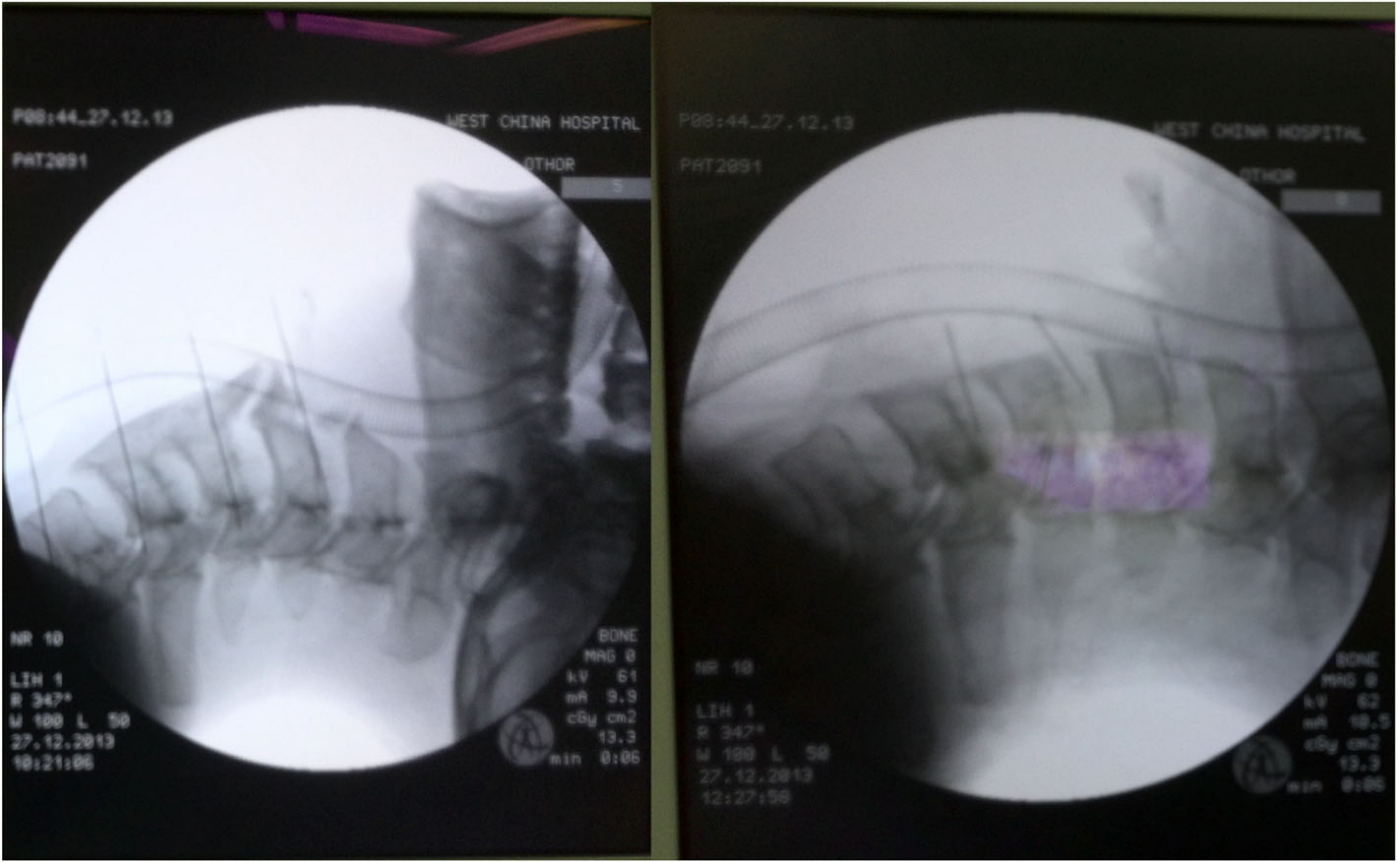
Preoperative and postoperative cervical spine radiography: endotracheal tube got across the “beak-like” osteophyte in front edges of the C4, C5 vertebrae before operation (left, a) and the beak-like osteophyte has disappeared after operation (right, b)

The location of the 7.5# enforced endotracheal tube was confirmed by a normal ETCO_2_, and symmetrical breathing sounds were heard from the lungs. The catheter depth was 22 cm from the central incisor. The endotracheal tube reached across the “beak-like” osteophyte in front of the C4–C5 vertebrae in the preoperative cervical spine radiograph (Fig. 3a). The “beak-like” osteophyte appeared resected in the postoperative cervical spine radiograph (Fig. 3b). The operation was successfully completed and lasted approximately 4 h. Then, the patient was transferred to the intensive care unit with the endotracheal tube retained in case of airway obstruction induced by postoperative laryngeal and tracheal edema. He was extubated after 1 day and discharged without any complications after 11 days of treatment. At the time of discharge, there was no numbness in the hands or walking instability. Moreover, there were no complications during a 12-month follow-up period after the surgery.

## Literature review

We systematically searched PubMed, EMBASE, and the Cochrane Library for records dated from inception to February 2020 and identified articles reporting anesthetic techniques for difficult airway in patients with OALL of the cervical spine. A comprehensive search strategy was employed using relevant search terms selected from the Medical Subject Headings, EmTree, and Entry terms. The search terms were as follows: (Hyperostosis, Diffuse Idiopathic Skeletal OR Diffuse Idiopathic Skeletal Hyperostosis OR Vertebral Ankylosing Hyperostosis OR Forestier’s Disease OR Forestier Rotes Disease OR Forestier Disease OR Calcification of Anterior Longitudinal Ligament OR calcific anterior longitudinal ligament OR Anterior Longitudinal Ligament Calcification OR Anterior Longitudinal Ligament Ossification OR Ossification of Anterior Longitudinal Ligament OR OALL OR cervical osteophytes OR cervical osteophytosis) AND (airway management OR difficult intubation OR difficult laryngoscopy OR difficult airway OR failed tracheal intubation OR difficult tracheal intubation). The search language was limited to English, and a total of 70 articles were retrieved. After removing duplicates, a total of 59 titles and abstracts were screened for eligibility. Of these, 34 full-text articles were evaluated, and 23 papers were potential candidates. One article was excluded because the full text could not be found [7], leaving 22 articles (summarized in Table 1) [5, 6, 8–27]. The excluded articles are presented in the appendix.

**Table 1:** Review of anesthetic techniques reported for patients with OALL of the cervical

Author and (year)	Age	Sex	Anesthesia Method	Intubation tube	Symptom	Osteophyte
Lee (1979) [5]	73	M	awake intubation	direct laryngoscope with Miller blade	asymptomatic	C5-C7
Gorback (1991) [8]	61	M	rapid induction	bullard laryngoscope	restricted motion of the head and neck	NA
Crosby (1993) [6]	71	M	rapid induction	direct laryngoscope	asymptomatic	C5-C6
Togashi (1993) [9]	59	M	rapid induction	direct laryngoscopy	restricted motion of the neck	C5-C7
Broadway (1994) [10]	72	F	NA	laryngeal mask airway	asymptomatic	C3-C4
Ranasinghe (1994) [11]	72	F	awake intubation	fiberscope	dysphagia,	C2-C4
Aziz (1995) [12]	68	M	sedation and analgesia	facemask airway	asymptomatic	C3-C5
Palmer(2000) [13]	48	F	awake intubation.	intubating laryngeal mask and fiberscope	dysphagia and restricted motion of the neck	C3
Bougak (2004) [14]	62	M	awake intubation.	fiberscope	asymptomatic	C3-C7
Naik (2004) [15]	55	M	awake intubation	fiberscope	restricted motion of the neck, dysphagia, obstructive sleep apnea, and dysphagia	C2-C6
Cesur (2005) [16]	57	M	rapid induction	direct laryngoscopy with Magill’s forceps	restricted motion of the neck	C2-C3
Ozkalkanli (2006) [17]	68	M	rapid induction	direct laryngoscope	restricted motion of the neck, dysphagia, dysphonia, and dyspnea	C2-C5
Montinaro (2006) [18]	67	M	NA	optical fibers	dysphagia, dysphonia	C3-C5
Satomoto (2007) [19]	67	M	NA	direct laryngoscope with the bougie guidance	dysphagia	NA
Baxi (2010) [20]	54	M	awake intubation	fiberoptic bronchoscope	dysphagia	C2-C3, C6-C7, T1
Thompson (2010) [21]	65	M	rapid induction	laryngeal mask airway and fibreoptic bronchoscope	asymptomatic	C3-C7
Eipe (2013) [22]	69	M	awake intubation	fibreoptic bronchoscope	dysphagia	C3-C5
Iida (2015) [23]	82	M	rapid induction	direct laryngoscope	dysphagia, aspiration pneumonia	C2-C4, C6-C7
Iida (2015) [23]	69	M	awake intubation	fibreoptic	restricted motion of the neck	C2-C3
Alsalmi (2018) [24]	66	M	awake intubation	fibreoptic bronchoscope	dysphagia, odynophagia, hoarseness	C3-C7
Gosavi (2018) [25]	62	M	awake intubation	fiberoptic bronchoscope	restricted motion of the neck, dysphagia, odynophagia	C2-C7
Garcia Zamorano (2019) [26]	85	M	sedation	fiberoptic bronchoscope	acute airway obstruction	C2-C5
Yoshimatsu (2019) [27]	80	M	NA	fiberoptic bronchoscope	sudden-onset upper airway obstruction, dysphonia, restricted motion of the neck	C2-C7

*OALL* Ossification of the anterior longitudinal ligament, *NA* Not available

A total of 23 patients with OALL of the cervical had a difficult airway. Only two patients were women [11, 13], and only one patient was younger than 50 years [13]. Previous epidemiological studies have suggested that the prevalence of OALL increases with age, and the morbidity rate was found to be significantly higher for men than for women [28]. Among the patients included, the most commonly involved cervical vertebrae were C3–C4, followed by C4–C5 and C5–C6, leading to dysphagia and airway obstruction, possibly due to excessive activity. Six patients had no symptoms before intubation [5, 6, 10, 12, 14, 21], and the rest of the patients had symptoms such as dysphagia, dysphonia, dyspnea, airway obstruction, or restricted motion of the neck [8, 9, 11, 13, 15–20, 22–27]. Awake intubation was chosen for 10 patients [5, 11, 13–15, 20, 22–25], and rapid induction was chosen for 7 patients [6, 8, 9, 16, 17, 21, 23]; fiberscope-assisted intubation was cited as the optimal choice in 13 articles [11, 13–15, 18, 20–27]; other cases favored the direct laryngoscope [5, 6, 9, 16, 17, 19, 23] or the intubating laryngeal mask [13, 21]. A small-sized endotracheal tube was selected for 4 patients [6, 17, 25, 27], while a nasotracheal tube was selected for 2 patients [11, 22]. The majority of patients required multiple endotracheal intubation attempts, and four patients could not undergo the surgery because of intubation failure [5, 14, 15, 18]. A laryngeal mask airway was used in one patient [10], a facemask airway was used in one patient [12], and thyrocricoid puncture and retrograde intubation were attempted in one patient [16]. We also identified nine cases of emergency tracheotomies due to sudden upper airway obstruction induced by OALL of the cervical spine [18, 28–35].

## Discussion and conclusion

Our literature review revealed that a difficult airway can be found in symptomatic [7, 8, 10, 12, 14–19, 21–26] and asymptomatic [5, 6, 10, 12, 14, 21] patients with OALL of the cervical spine who require surgery. Therefore, this possibility should be considered by anesthesiologists treating symptomatic patients with OALL and, as presented in this case report, those with cervical disease combined with asymptomatic OALL. Our radiography and MRI findings revealed OALL of the cervical spine, with prominent osteophytes involving four cervical vertebrae in combination with a bulge in the posterior throat wall, and a narrow pharyngeal space. This, with the inability to visualize the glottis, resulted in a difficult airway. The imaging data could suggest that the patient was at risk for difficult intubation. A postmortem study revealed that hypertrophic osteophytes were present in the cervical spines of 21 out of 75 asymptomatic patients (28%) during autopsy [36]. Cervical spine radiography is not routinely performed when patients with asymptomatic OALL of the cervical spine requires the performance of other surgeries or when symptomatic patients conceal their condition before surgery. Furthermore, it is difficult to recognize the risk of difficult intubation in such patients, despite routine preoperative evaluations for anesthesia. Therefore, to prevent challenges faced during an unanticipated difficult intubation, anesthesiologists should consider the possibility of a difficult airway in symptomatic and asymptomatic patients with OALL of the cervical spine.

Although appropriate guidelines are available for the management of unanticipated difficult intubation [37], unexpected difficult airways continue to concern anesthesiologists and endanger patients. According to our literature search, an unexpected difficult airway induced by OALL of the cervical spine leads to termination of the operation [5, 14, 15, 18]. In one case of a distorted airway caused by osteophytes, fiberoptic nasal intubation was extremely difficult, and an emergency tracheotomy had to be performed [11]. Therefore, to ensure the patient’s safety, difficult airways induced by OALL of the cervical spine should be identified before surgery.

A critical question is how can we predict the possibility of a difficult airway induced by OALL of the cervical spine? Although radiological evaluation may be useful in assessing the risk of difficult intubation, it is still not recommended because OALL of the cervical spine is a relatively common condition that is only occasionally associated with difficult intubation [14]. Currently, the etiology and pathogenesis of OALL remain unclear, but this condition is strongly associated with frequently diagnosed metabolic abnormalities and joint degeneration [1]. In addition, it may be related to increased cervical motion or trauma. A recommendation to screen patients with risk factors, which should make the anesthesiologist suspect a difficult airway, should be entertained. Our literature review noted that men were more commonly affected than women, the disease was rare in patients younger than 50 years, and the incidents became more common as the age was advanced [38]. Patients with obesity, hypertension, diabetes, dyslipidemia, hyperuricemia, neck injury, cervical surgery history, osteoarthritis, ossification of the posterior longitudinal ligament and Forestier’s disease, or DISH were more likely to have cervical OALL [39, 40]. In these cases, cervical radiography and a detailed evaluation of the range of neck motion and swallowing function should be emphasized. Additionally, more effective clinical evaluation methods should be determined.

**Fig. 4:**
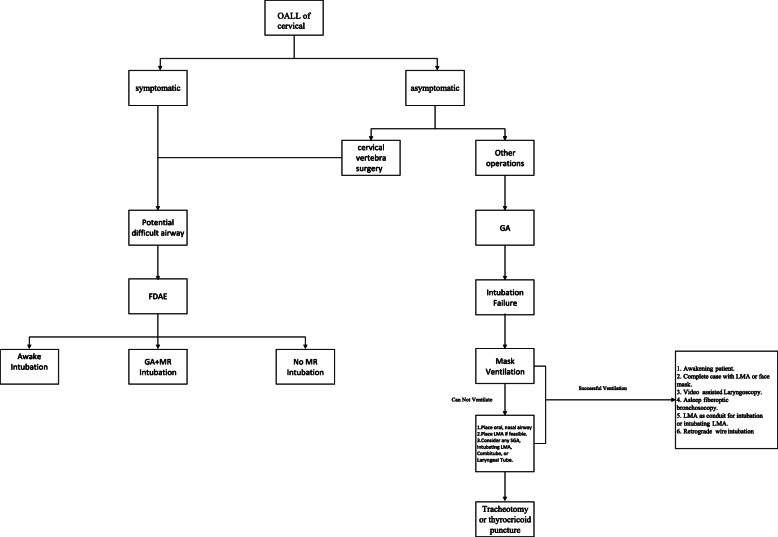
A summary of intubation methods in patients with OALL of the cervical. FADE, fast difficult airway evaluation; GA, general anesthesia; MR, muscle relaxants; LMA, laryngeal mask airway

Moreover, our literature review found that in patients with OALL of the cervical spine with an anticipated difficult intubation, a fiberoptic bronchoscope-assisted awake intubation was the optimal method of intubation. The methods of intubation in patients with OALL of the cervical spine are summarized in Fig. 4. In general, a difficult airway was caused by limitations in cervical mobility and airway obstruction caused by OALL. Normally, the larger the osteophytes, the more evident the clinical presentations, and a difficult airway induced by osteophytes could also cause more severe symptoms. Therefore, routine radiological evaluation is important to determine the airway status in patients with OALL of the cervical spine and should be emphasized during preoperative anesthesia visits, especially for patients with airway obstructions, hoarseness, or other symptoms. It is beneficial to evaluate the degree of ossification and its impact on the surrounding tissue to identify the risk of a difficult intubation. Then, the physicians can strategize and arrange for the appropriate equipment. We recommend a fast-difficult airway evaluation in patients with potentially difficult ventilation/difficult intubation [41]. In brief, patients should gradually be sedated with sevoflurane, and the adequacy of manual mask ventilation during spontaneous breathing should be assessed at various sedation levels. Awake intubation with the Airtraq® video-laryngoscope or fiberoptic bronchoscope can be applied in cases with inadequate mask ventilation and severe airway obstruction. When adequate mask ventilation is retained and the vocal cords are visible, the patient can be intubated under general anesthesia.

When asymptomatic patients with OALL face an unanticipated difficult intubation, anesthesiologists should be aware of the possibility of a difficult airway due to OALL of the cervical spine and should follow the unanticipated difficult airway guidelines. Most importantly, adequate ventilation should be maintained through oropharyngeal, nasopharyngeal, or laryngeal mask airways. Then, the intubation equipment can be chosen after an airway assessment, using a direct laryngoscope, such as the UE® and the Airtraq® video laryngoscopes or a fiberoptic bronchoscope. In particular, a laryngoscopy using Airtraq® may alter the Cormack-Lehane score from III or IV to I or II. An emergency tracheotomy or thyrocricoid puncture can be performed where necessary.

In conclusion, it is important for anesthesiologists and spine surgeons to be aware and be prepared for the possibility of a difficult airway induced by OALL of the cervical spine. In case of an unanticipated difficult intubation, the anesthesiologist should be able to refer to the unanticipated difficult airway guidelines and identify OALL of the cervical spine as the cause of the difficult airway.

## Supplementary information

**Additional file 1: Supplemental Text 1.** Excluded articles after review of full text and reasons for their exclusion.

## Data Availability

Not applicable.

## Abbreviations

OALL: Ossification of the anterior longitudinal ligament
MRI: Magnetic resonance imaging
