# Supplementary material for: Ossification of the cervical anterior longitudinal ligament is an underdiagnosed cause of difficult airway: a case report and review of the literature
Source: BMC Anesthesiol. 2020 Jun 30;20:161. doi: 10.1186/s12871-020-01077-9 (PMC7325286; doi:10.1186/s12871-020-01077-9)
Supplement: Supplementary file 1 — Additional file 1: Supplemental Text 1. Excluded articles after review of full text and reasons for their exclusion. [file 12871_2020_1077_MOESM1_ESM.doc]

**Supplemental Text 1. Excluded articles after review of full text and reasons for their exclusion.**

Reasons for exclusion (numbers denote the reference number below)

1. Unaffiliated of this study after screening the titles and abstracts [1-25]
2. Conference abstract [26, 27]
3. Full text not available [28]
4. Imaging report[29]
5. Emergency tracheotomies due to sudden upper airway obstruction [30-37]

1. Hassard AD: **Cervical ankylosing hyperostosis and airway obstruction**. *The Laryngoscope* 1984, **94**(7):966-968.

2. Halama AR: **Surgical treatment of oropharyngeal swallowing disorders**. *Acta oto-rhino-laryngologica Belgica* 1994, **48**(2):217-227.

3. Kingdom TT, Nockels RP, Kaplan MJ: **Transoral-transpharyngeal approach to the craniocervical junction**. *Otolaryngology--head and neck surgery : official journal of American Academy of Otolaryngology-Head and Neck Surgery* 1995, **113**(4):393-400.

4. Barbe F, Pons S, Togores B, Sauleda J, Soler R, Agusti AG: **Continuous positive airway pressure is effective in treating upper airway oedema**. *The European respiratory journal* 1996, **9**(5):1092-1093.

5. Krnacik MJ, Heggeness MH: **Severe angioedema causing airway obstruction after anterior cervical surgery**. *Spine* 1997, **22**(18):2188-2190.

6. Barclay K, Asai T: **Laryngeal function may be impaired in patients with cervical osteophytes**. *European journal of anaesthesiology* 1998, **15**(2):250-251.

7. Epstein NE: **Circumferential surgery for the management of cervical ossification of the posterior longitudinal ligament**. *Journal of spinal disorders* 1998, **11**(3):200-207.

8. Das A, Sivak MV, Jr., Chak A: **Cervical esophageal perforation during EUS: a national survey**. *Gastrointestinal endoscopy* 2001, **53**(6):599-602.

9. Dickerman RD, Zigler JE: **Atraumatic vertebral artery dissection after cervical corpectomy: a traction injury?** *Spine* 2005, **30**(21):E658-e661.

10. Ahlawat SK, Haddad N: **Repair of an EUS - Induced duodenal perforation with endoscopic clips**. *Acta gastro-enterologica Belgica* 2009, **72**(3):361-364.

11. Miake G, Arizono T, Yamaguchi T, Akune H: **Case of critical complication after surgery for ankylosing spinal hyperostosis**. *Fukuoka igaku zasshi = Hukuoka acta medica* 2009, **100**(4):104-107.

12. Goh PY, Dobson M, Iseli T, Maartens NF: **Forestier's disease presenting with dysphagia and dysphonia**. *Journal of clinical neuroscience : official journal of the Neurosurgical Society of Australasia* 2010, **17**(10):1336-1338.

13. Kurata Y, Yoshimoto M, Takebayashi T, Kawaguchi S, Yamashita T: **Subarachnoid-pleural fistula treated with noninvasive positive pressure ventilation: a two-case report and literature review**. *Spine* 2010, **35**(18):E908-911.

14. Anand V, Vikram Vel VR, Purushothaman PK, Rajesh Kumar MS: **Crico Arytenoid Joint Fixation in Diffuse Idiopathic Skeletal Hyperostosis (DISH): A Case Report**. *Indian journal of otolaryngology and head and neck surgery : official publication of the Association of Otolaryngologists of India* 2011, **63**(Suppl 1):55-57.

15. Carlson ML, Archibald DJ, Graner DE, Kasperbauer JL: **Surgical management of dysphagia and airway obstruction in patients with prominent ventral cervical osteophytes**. *Dysphagia* 2011, **26**(1):34-40.

16. Eyigor H, Selcuk OT, Osma U, Koca R, Yilmaz MD: **Cervical osteophytes: a rare cause of obstructive sleep apnea**. *The Journal of craniofacial surgery* 2012, **23**(5):e444-446.

17. Kawauchi E, Yamagata T, Tohda Y: **A case of Forestier disease with obstructive sleep apnea syndrome**. *Sleep & breathing = Schlaf & Atmung* 2012, **16**(3):603-605.

18. Mermigkis C, Mermigkis D, Bouloukaki I, Alogdianakis V, Schiza SE: **Hooking of the soft palate and a large cervical osteophyte: two troubles in the same airway**. *The American journal of the medical sciences* 2013, **346**(6):519-520.

19. Bo L, Li J, Tao T, Bai Y, Ye X, Hotchkiss RS, Kollef MH, Crooks NH, Deng X: **Probiotics for preventing ventilator‐associated pneumonia**. *Cochrane Database of Systematic Reviews* 2014(10).

20. von der Hoeh NH, Voelker A, Jarvers JS, Gulow J, Heyde CE: **Results after the surgical treatment of anterior cervical hyperostosis causing dysphagia**. *European spine journal : official publication of the European Spine Society, the European Spinal Deformity Society, and the European Section of the Cervical Spine Research Society* 2015, **24 Suppl 4**:S489-493.

21. Gill JR, Morrissey DI, Van Rensburg L, Tytherleigh-Strong G: **Sternoclavicular joint osteophytosis: a difficult diagnosis to swallow**. *BMJ case reports* 2017, **2017**.

22. Sebaaly A, Boubez G, Sunna T, Wang Z, Alam E, Christopoulos A, Shedid D: **Diffuse Idiopathic Hyperostosis Manifesting as Dysphagia and Bilateral Cord Paralysis: A Case Report and Literature Review**. *World neurosurgery* 2018, **111**:79-85.

23. Dell'Era V, Garzaro M, Farri F, Gorris C, Rosa MS, Toso A, Aluffi Valletti P: **Respiratory presentation of diffuse idiopathic skeletal hyperostosis (DISH): Management and review of the literature**. *Cranio : the journal of craniomandibular practice* 2019:1-4.

24. Guay J, Kopp S: **Epidural analgesia for adults undergoing cardiac surgery with or without cardiopulmonary bypass**. *Cochrane Database of Systematic Reviews* 2019(3).

25. Tariq R, Enslin S, Kaul V: **Pharyngo-esophageal perforation during endoscopic ultrasound: Case report and literature review**. *American Journal of Gastroenterology* 2019, **114 (Supplement)**:S1176-S1177.

26. Marques A, Nora D, Ferreira C: **When bones are on the (air)way: Giant anterior cervical osteophyte causing airway compromise**. *Anaesthesia* 2011, **1)**:9.

27. Eipe N, Fossey S, Kingwell S: **The ankylosing spondylitis airway-between a rock and a hard place**. *Canadian Journal of Anesthesia* 2011, **1)**:S125.

28. Thapa D, Sinha PK, Gombar S, Gombar KK, Palta S, Sen I: **Large anterior cervical osteophytes: A cause for laryngeal "BURP" failure and difficult intubation - A case report**. *Journal of Anaesthesiology Clinical Pharmacology* 2002, **18**(3):320-322.

29. Fujii M, Sugiyama D, Ueda K, Kobayashi O: **Forestier Disease and Airway Management**. *Anesthesiology* 2020.

30. Psychogios G, Jering M, Zenk J: **Cervical Hyperostosis Leading to Dyspnea, Aspiration and Dysphagia: Strategies to Improve Patient Management**. *Frontiers in surgery* 2018, **5**:33.

31. Hoey AW, Dusu K, Gane S: **Diffuse idiopathic skeletal hyperostosis (DISH): an unusual cause of airway obstruction**. *BMJ case reports* 2017, **2017**.

32. Bird JH, Biggs TC, Karkos PD, Repanos C: **Diffuse idiopathic skeletal hyperostosis as an acute airway presentation requiring urgent tracheostomy**. *The American journal of emergency medicine* 2015, **33**(5):737.e731-732.

33. Varsak YK, Eryilmaz MA, Arbag H: **Dysphagia and airway obstruction due to large cervical osteophyte in a patient with ankylosing spondylitis**. *The Journal of craniofacial surgery* 2014, **25**(4):1402-1403.

34. Lin HW, Quesnel AM, Holman AS, Curry WT, Jr., Rho MB: **Hypertrophic anterior cervical osteophytes causing dysphagia and airway obstruction**. *The Annals of otology, rhinology, and laryngology* 2009, **118**(10):703-707.

35. Caminos CB, Cenoz IZ, Louis CJ, Otano TB, Esain BF, Perez de Ciriza MT: **Forestier disease: an unusual cause of upper airway obstruction**. *The American journal of emergency medicine* 2008, **26**(9):1072.e1071-1073.

36. Matan AJ, Hsu J, Fredrickson BA: **Management of respiratory compromise caused by cervical osteophytes: a case report and review of the literature**. *The spine journal : official journal of the North American Spine Society* 2002, **2**(6):456-459.

37. Demuynck K, Van Calenbergh F, Goffin J, Verschakelen J, Demedts M, Van de Woestijne K: **Upper airway obstruction caused by a cervical osteophyte**. *Chest* 1995, **108**(1):283-284.
